# Different Surgical Strategy for Patients with Cervical Angina: A Potential Role of Luschka's Joint Osteophyte

**DOI:** 10.1111/os.12751

**Published:** 2020-08-23

**Authors:** Fan Feng, Xiu‐yuan Chen, Long Shen, Quan Li, Li‐feng Lao, Hong‐xing Shen

**Affiliations:** ^1^ Department of Spine Surgery Renji Hospital, Shanghai Jiaotong University School of Medicine Shanghai China; ^2^ Department of Cardiology Renji Hospital, Shanghai Jiaotong University School of Medicine Shanghai China

**Keywords:** Anterior cervical decompression, Cervical angina, Luschka's joint osteophyte

## Abstract

**Objective:**

Cervical angina is an underrecognized type of noncardiac chest pain and its mechanism of pain remains obscure. The objective of the current study was to investigate the clinical outcomes of different surgical strategies for patients with cervical angina and to analyze the potential pathogenesis of Luschka's joint osteophyte.

**Methods:**

From February 2013 to March 2018, a prospective study on cervical angina was performed in our hospital. All patients who were diagnosed with both noncardiac chest pain and cervical pathology were identified. During admission, they consulted with a cardiologist and underwent strict cardiac workups to exclude true angina pectoris. The included 41 patients were randomly divided into two groups according to different surgical strategies of whether or not to remove Luschka's joint osteophyte during anterior cervical decompression surgery: the osteophyte resection (OR group) and the nonresection (NR group).

**Results:**

The OR group consisted of 21 patients (8 men and 13 women) with a mean age of 54.7 years (range, 41–65 years). The NR group was composed of 20 patients (9 men and 11 women) with an average age of 56.3 years (range, 43–68 years). Before surgery, the mean duration of symptoms was 6.1 months (range, 4–20 months). The Luschka's joint osteophytes were located at C_6_–C_7_ (19 cases, 46.3%), C_5_–C_6_ (17 cases, 41.5%), and C_4_–C_5_ (4 cases, 12.2%). Their average area was 34.85 mm^2^ and the average length were 5.09 mm. No statistically significant differences in demographic characteristics were detected between the two groups (*P* > 0.05). After operation, there were significant improvements in the Japanese Orthopedic Association score and the Neck Disability Index score in both groups (*P* < 0.05). However, the visual analogue scale score for chest pain in the OR group was statistically lower than that in the NR group (1.4 ± 1.0 *vs* 2.1 ± 1.6, *P* < 0.05). In the OR group, the results of cervical spine surgery were excellent in 18 patients (85.7%), and fair in 3 patients (14.3%). In the NR group, there were 10 patients (50.0%) with excellent results, 9 patients with fair results (45.0%), and 1 patient with poor results (5.0%). Notably, there were statistically significant differences between the two groups (χ^2^ = 6.265, *P* = 0.044). The average follow‐up was 31 months (24–52 months).

**Conclusion:**

Anterior cervical decompression surgery with resection of Luschka's joint osteophyte can effectively reduce cervical angina symptom and improve the patient's quality of life. In addition to nerve root compression, Luschka's joint osteophyte may be another pathogenic factor in cervical angina.

## Graphical abstract

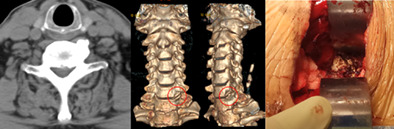

## Introduction

Chest pain is a common and highly challenging clinical problem in emergency departments. However, only 15% to 25% of patients with acute chest pain actually have acute coronary syndrome^1^. The majority of patients underwent cardiac “rule out” and were diagnosed with noncardiac chest pain. Noncardiac chest pain occurs in various gastrointestinal diseases, but it can also be caused by skeletal conditions. Cervical angina is a kind of chest pain resembling true cardiac angina but originates from disorders of the cervical spine^1^, ^2^.

Although many previous investigators have described this condition^3^, ^4^, ^5^, ^6^, cervical angina still appears to be neglected in routine clinical practice. Iwasa^7^ found that only 4.7% of patients with angina pectoris had cervical nerve root compression. Nakajima *et al*.^8^ reviewed 706 patients who underwent cervical spine surgeries and found that 1.4% of those were considered cervical angina. Patients with cervical angina often present with anterior chest pain. The pain has been described as sharp, achy, or crushing in quality. Symptoms may be present at rest or exacerbated by physical activity. More than half of patients experience autonomic symptoms, such as dyspnea, vertigo, nausea, fatigue, and headaches^2^, ^4^, ^8^.

Cervical angina appears to be a relatively unknown clinical syndrome compared with other angina symptoms. The neurological symptoms are often neglected. Prompt diagnosis of this underrecognized disorder requires an awareness of the common presenting features of cervical angina and requires a strong sense of suspicion in patients with inadequately explained chest pain. Varying degrees of cardiac workups were performed to exclude true angina pectoris. Conventional criteria, such as response to nitroglycerin and stress electrocardiogram, all carry a high percentage of error. When coronary disease fails to explain the patient's angina pain, it is quite natural for one to think first of functional overlay and to tend to attribute the pain to a psychosomatic cause. This is understandable because most patients with angina pain of moderate duration, especially if they had a normal coronary angiography, do, indeed, manifest nervousness and anxiety about the condition. However, most such patients have chest wall pain due to organic systems capable of reproducing angina, such as cervical spine or gastrointestinal disorders, before settling on a psychosomatic basis as the only diagnosis^2^. Patients with cervical angina remain underdiagnosed, untreated, and potentially disabled in terms of anxiety, depression, and activities of daily living.

However, the mechanism of chest pain is still controversial. Most investigators believe cervical angina is a kind of radicular pain, secondary to root compression by a herniated disc, osteoarthritic spurs, or compression in a narrow intervertebral foramen^1^, ^2^, ^3^, ^7^, ^9^. Divisions of the anterior roots of C_5_ to T_1_ give origin to a number of individual nerves that supply motor, sensory, and autonomic fibers to the upper thorax, shoulder, and arm^1^. The efficacy of symptoms relief following cervical discectomy and fusion, in return, proves the theory of nerve root compression in cervical angina^2^, ^4^. Some cases have also been reported in which cervical angina was attributed to cervical myelopathy^8^, ^10^, spinal cord infarction^11^, spinal cord tumors^12^, cervical instability^13^, and intervertebral disc disease^9^.

Conservative treatment such as the use of head traction or a neck collar could help in pain elimination, either transiently or permanently^14^. However, in cases where neurological compromise is evident or conservative therapy fails, surgical intervention should be indicated to achieve rapid improvement and better quality of life^2^, ^4^, ^8^. Brodsky^2^ reported the largest series of 438 cases with cervical angina; 88 of them (20.1%) were surgically treated, mostly by anterior approach. Nakajima *et al*.^8^ and Ozgur *et al*.^9^ also demonstrated that chest pain relief were achieved by anterior cervical surgery decompression. Anterior cervical surgery to correct nerve root or spinal cord compression has proved to be a useful option for pain relief in cervical angina^13^.

In our clinical practice, we noticed that some patients with cervical angina have great Luschka's joint osteophyte in their degenerative cervical vertebrae, which may have close relationships with sympathetic afferent fibers to the heart and coronary arteries. The sympathetic trunk may be vulnerable to irritation because it is situated close to the medial border of the longus colli muscle at the lower cervical spine region. We hypothesize that the protrusion of Luschka's joint osteophyte jacks up the homolateral longus colli, which might compress or stimulate adjacent sympathetic afferent fibers to the heart and coronary arteries and result in noncardiac chest pain. It is worth investigating whether these patients’ pseudoangina could be resolved after anterior cervical surgery with resection of Luschka's joint osteophyte.

Therefore, this prospective study evaluated the clinical outcome of surgical treatment for cervical angina patients as compared with those without resection of Luschka's joint osteophyte. The objective of the current study was to: (i) raise awareness of these clinical symptoms in the diagnosis of cervical spine disorders; (ii) provide surgeons with practical experiences on different surgical strategy for cervical angina patients regarding whether resection of Luschka's joint osteophyte; and (iii) analyze the radiographic characteristics of Luschka's joint osteophyte and its potential pathogenesis in cervical angina.

## Materials and Methods

### *Subjects*

In February 2013, after obtaining approval from the institutional review board at our hospital, we initiated a prospective study of patients with cervical angina. For inclusion, all patients who were diagnosed with both noncardiac chest pain and cervical spondylosis were identified.

The inclusion criteria were as follows: (i) patients presenting with chest pain who consulted with a cardiologist during the admission and then underwent strict cardiac workups to exclude the true angina pectoris; (ii) chest pain was not relieved by conservative treatment for at least 3 months; (iii) MRI findings of cervical pathology (cervical radiculopathy or myelopathy); (iv) CT exhibited Luschka's joint osteophytes on degenerated cervical spine; and (v) patients underwent cervical surgery with a minimum of 2 years of follow up. The exclusion criteria were as follows: (i) cervical spine tumor; (ii) cervical infection (specific/non‐specific); (iii) cervical spine trauma; and (iv) combination of heart disease.

Through March 2018, a total of 41 patients fulfilling the inclusion criteria were recruited into this study. There were 17 male and 24 female patients, with an average age of 55.5 years (41–68 years) at surgery. Of these, 32 patients visited our hospital directly because of typical cervical‐related symptoms associated with precordial pain, while the clinical presentations of the other 9 cases were first misdiagnosed as acute coronary syndrome in the emergency room. During the hospitalization, each patient consulted with a cardiologist to rule out coronary artery disease. All patients had an electrocardiogram and a cardiac enzymes test, which were unremarkable. A total of 27 patients underwent coronary angiography during prior admissions. All coronary angiograms performed had normal results.

**Fig. 1:**
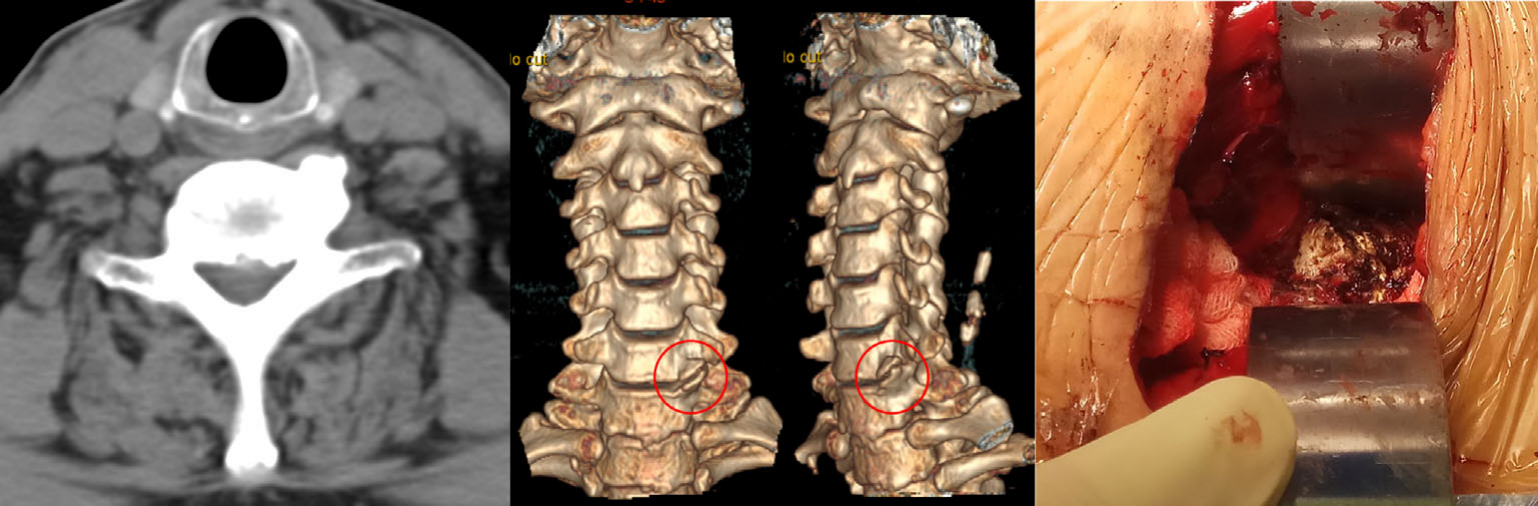
Cervical CT scans demonstrate the Luschka's joint osteophyte in patient's degenerative cervical vertebrae (red circle); the exposure and resection of Luschka's joint osteophytes during the anterior cervical decompressive surgery.

To evaluate the potential role of Luschka's joint osteophyte, the patients were randomly divided into two groups according to different surgical strategies of whether to remove Luschka's joint osteophyte during anterior cervical decompression surgery: an osteophyte resection (OR) group and a nonresection (NR) group. All surgeries were performed by the same senior surgeon using a uniform surgical technique as described previously^15^, ^16^, ^17^. A right‐side anterolateral transverse incision was pursued for the anterior cervical spine followed by Robinson's anterior decompression and interbody fusion. The posterior longitudinal ligament and fragments of the disk were removed to complete decompression of the spinal cord. In the OR group, subperiosteal colli longus was dissected and Luschka's joint osteophytes were exposed. Then, the hypertrophic osteophyte was removed using bone rongeur and high speed drill (Fig. 1). While in the NR group, the Luschka's joint osteophytes were reserve *in situ*.

### *Radiographic Measurements*

**Fig. 2:**
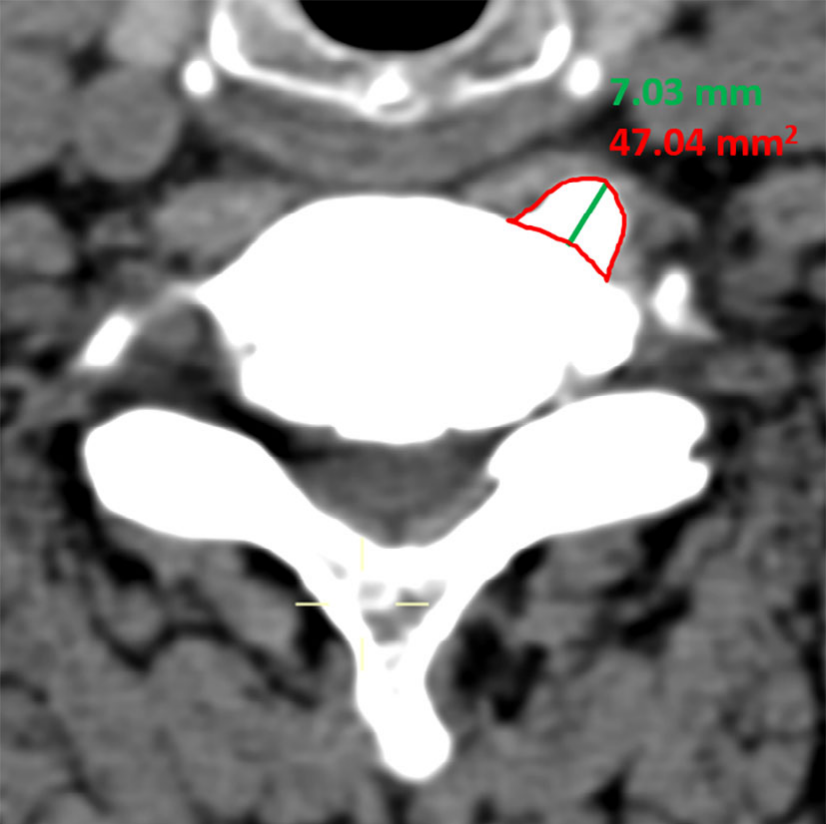
Radiographic measurements: Area of Luschka's joint osteophytes (red boundary), length of Luschka's joint osteophyte (green line).

Cervical CT scans (light speed VCT; GE Medical Systems, Milwaukee, WI) were obtained before surgery. Given that the orientation of CT cuts would change the measurements, the axial images used for the measurement were selected by referencing the sagittal image and viewing the cut that included both the largest cross‐sectional area of Luschka's joint osteophytes and paralleled to the upper endplate of vertebrae. Subsequently, the following two parameters were measured (Fig. 2).

#### *Area of Luschka's Joint* *Osteophyte*

The area of Luschka's joint osteophyte (mm^2^) was defined as the boundary of Luschka's joint osteophyte on the axial image of CT scan. The area of Luschka's joint osteophyte was measured to determine the maximum dimension of the osteophyte and to evaluate the degree of this osteophyte compressing on the homolateral longus colli. We also analyzed whether there was any difference of area of Luschka's joint osteophyte between two groups.

#### *Length of Luschka's Joint Osteophyte*

The length of Luschka's joint osteophyte (mm) was defined as the largest distance between the top of Luschka's joint osteophyte and the anterior edge of the vertebral body. The length of Luschka's joint osteophyte was measured to evaluate the hypertrophy of Luschka's joint and the degree of osteophyte compressing on homolateral longus colli. The length of Luschka's joint osteophyte was compared between the two groups.

The radiological data were analyzed using PACS client software (version 11.4; Carestream health). All of the measurements were performed by an independent observer.

### *Evaluation of Surgical Outcomes*

According to the method of Brodsky^2^, surgical outcomes of cervical spine surgery for cervical angina were classified into three grades: (i) excellent, relief of preoperative chest pain; (ii) fair, occasionally mild chest pain requiring no medications; and (iii) poor, continued same or less severe chest pain, requiring occasional medication. Moreover, all patients’ preoperative *versus* postoperative neurologic function and clinical status was routinely evaluated using a scoring system: the Japanese Orthopedic Association (JOA) score, the neck disability index (NDI) score, and the pain visual analog scale (VAS) score. Specifically, in this series, the VAS score only referred to a patient's chest pain, and not the upper extremity radiating pain.

#### *Japanese Orthopedic Association*

The JOA^18^ was used to evaluate the spinal cord function, which was assessed preoperatively and at last follow‐up. The total score of the JOA was 17 points, including upper limb motor function (four points), lower limb motor function (four points), upper limb sensory function (two points), trunk sensory function (two points), lower limb sensory function (two points), and bladder function (two points).

#### *Neck Disability Index*

The NDI^19^ was used to evaluate neck pain and disability. The NDI contains 10 self‐reported items, including: pain intensity, personal care, lifting, reading, headache, concentration, working, sleeping, driving, and entertainment. Each item is scored from zero to five. The final score was presented as the percentage of the maximal score. The final NDI score is calculated as (total score/(five × number of questions answered)) × 100%, where 0% to 20% is considered mild dysfunction, 21%–40% is moderate dysfunction, 41%–60% is severe dysfunction, and 61%–80% is considered as disability; 81% percent to 100% is either long‐term bedridden or the patient is exaggerating the impact of pain on their life.

#### *Visual Analogue Scale*

The VAS score system^20^ is used in the social and behavioral sciences to measure low back pain and leg pain. The VAS pain scoring standard (scores from 0 to 10) was as follows: 0 means painless; 1–3 means mild pain that the patient could endure; 4–6 means the patient was in pain that could be endured and be able to sleep; and 7–10 means the patient had intense pain and was unable to tolerate the pain.

### *Statistical Analysis*

All the statistical analysis was performed with SPSS software (version 21.0; SPSS, Chicago, IL). The comparison of questionnaire scores between preoperatively and the last follow up were performed with the Student *t*‐test. Comparisons of categorical variables were calculated by the χ^2^‐test. Moreover, the Pearson correlation coefficient was used to evaluate the association between the radiographic measurements and clinical questionnaires. Significance was defined as a *P*‐value of less than 0.05.

## Results

### *Preoperative Characteristics*

The OR group consisted of 21 patients (8 men and 13 women) with a mean age of 54.7 years (range, 41–65 years). The NR group was composed of 20 patients (9 men and 11 women) with the average age of 56.3 years (range, 43–68 years). The demographic characteristics of patients are presented in Table 1. Chest pain was located at the precordium in 24 cases, the retrosternal region in 10 cases, and the substernal area in 7 cases. These symptoms could be relieved by conservative treatments (i.e. bed rest, cervical traction, and non‐steroidal anti‐inflammatory drugs) for at least 3 months. Before surgery, the mean duration of symptoms was 5.9 months (range: 4–18 months) in the OR group and 6.4 months (range: 5–20 months) in the NR group. There no statistically significant differences in demographic characteristics between the two groups (*P* > 0.05) (Table 1).

**TABLE 1:** Demographic characteristics in two groups with cervical angina

Characteristics	OR group	NR group	*P‐*value
	(21 cases)	(20 cases)	
Age (mean ± SD, years)	54.7 ± 9.2	56.3 ± 8.0	0.271
Sex (M/F)	8/13	9/11	0.654
Location of chest pain			0.710
Precordium	11	13	
Retrosternal region	6	4	
Substernal area	4	3	
Duration of symptoms (mean ± SD, months)	5.9 ± 2.3	6.4 ± 2.1	0.639
Cervical MRI findings			0.645
Radiculopathy	17	15	
Myelopathy	4	5	
Affected levels*			0.873
C_3–4_	2	1	
C_4–5_	11	9	
C_5–6_	14	16	
C_6–7_	15	13	
Location of Luschka's joint osteophyte			0.702
C_4–5_	2	3	
C_5–6_	8	9	
C_6–7_	11	8	
Length of Luschka's joint osteophyte (mean ± SD, mm)	5.21 ± 1.63	4.98 ± 2.07	0.116
Area of Luschka's joint osteophyte (mean ± SD, mm^2^)	35.24 ± 8.22	34.46 ± 9.51	0.479

The osteophyte resection (OR) group consists of patients with resection of Luschka's joint osteophyte; the nonresection (NR) group consists of patients without resection of Luschka's joint osteophyte.

* Some cases had multiple affected levels.

Based on MRI findings, cervical spondylotic radiculopathy was found in 32 cases (78.0%), and cervical spondylotic myelopathy in 9 cases (22.0%). The affected levels were C_3–4_ level in 3 cases, C_4–5_ level in 20 cases, C_5–6_ level in 30 cases, and C_6–7_ level in 28 cases (some cases had multiple affected levels). The details of each group are presented in Table 1. In the NR group, the Luschka's joint osteophyte was located at C_6_–C_7_ (11 cases, 52.4%), C_5_–C_6_ (8 cases, 38.1%), and C_4_–C_5_ (2 cases, 9.5%). The average area of Luschka's joint osteophyte was 35.24 ± 8.22 mm^2^ and the mean length of Luschka's joint osteophyte was 5.21 ± 1.63 mm. In the NR group, the Luschka's joint osteophyte was located at C_5_–C_6_ (9 cases, 45.0%), C_6_–C_7_ (8 cases, 40.0%), and C_4_–C_5_ (2 cases, 15.0%).

### *Radiographic Measurements*

#### *Area of Luschka's Joint Osteophyte*

In the OR group, the average area of Luschka's joint osteophyte was 35.24 ± 8.22 mm^2^. In the NR group, the average area of Luschka's joint osteophyte was 34.46 ± 9.51 mm^2^. No statistical differences were detected between the two groups (*P* > 0.05).

#### *Length of Luschka's Joint Osteophyte*

The mean length of Luschka's joint osteophyte was 5.21 ± 1.63 mm in the OR group and 4.98 ± 2.07 mm in the NR group, respectively. There were no statistically significant differences between the two groups (*P* > 0.05).

To clarify the relationship between cervical angina and the characteristics of Luschka's joint osteophyte, the Pearson correlation coefficient was used to evaluate the association between the radiographic measurements and clinical questionnaires. However, no significant correlations were detected with regard to the results (Table 2).

**TABLE 2:** Results of the Pearson correlation analysis between the measurements of the Luschka's joint osteophyte and clinical questionnaires

Preoperative scores	Area of Luschka's joint osteophyte		Length of Luschka's joint osteophyte	
	*r* value	*P* value	*r* value	*P* value
JOA	−0.092	0.624	−0.113	0.435
NDI	0.275	0.740	0.138	0.362
VAS (thoracic pain)	0.633	0.071	0.391	0.067

JOA, Japanese orthopedic association; NDI, neck disability index; VAS, the pain visual analog scale.

### *Clinical Questionnaires of Patients with Cervical Angina*

Overall, the patients in this study achieved significant neurologic function and clinical outcome improvement after cervical surgeries (Table 3).

**TABLE 3:** The comparison of clinical questionnaire scores in two groups with cervical angina

Scores	OR group			NR group		
	Preoperation	Postoperation 1 week	Final follow‐up	Preoperation	Postoperation 1 week	Final follow‐up
JOA	10.1 ± 1.8	14.8 ± 1.1*	14.1 ± 1.2*	10.5 ± 2.2	14.3 ± 1.0*	14.4 ± 1.3*
NDI	32.5 ± 8.0	14.7 ± 5.9*	15.8 ± 7.1*	35.6 ± 10.3	15.4 ± 7.2*	16.3 ± 8.0*
VAS (chest pain)	5.5 ± 2.2	2.4 ± 1.5*	1.4 ± 1.0* †	5.3 ± 2.6	2.8 ± 1.9*	2.1 ± 1.6* †

JOA, Japanese orthopedic association; NDI, neck disability index; NR, the nonresection; OR, osteophyte resection; VAS, the pain visual analog scale.

* Statistical difference compared with preoperation (*P* < 0.05).

^†^ Statistical difference compared between the two groups (*P* < 0.05).

#### *Japanese Orthopedic Association Score*

The JOA score increased from 10.1 ± 1.8 to 14.8 ± 1.1 after the operation in the OR group and increased from 10.5 ± 2.2 to 14.3 ± 1.0 after the operation in the NR group. No statistical differences were observed between the two groups (*P* > 0.05).

#### *Neck Disability Index Score*

The NDI score decreased from 32.5 ± 8.0 to 14.7 ± 5.9 after the operation in the OR group and decreased from 35.6 ± 10.3 to 15.4 ± 7.2 after the operation in the NR group. There were no statistically significant differences between the two groups (*P* > 0.05).

#### *Visual Analog Scale Score*

The VAS score of chest pain decreased from 5.5 ± 2.2 to 2.4 ± 1.5 in the OR group and decreased from 5.3 ± 2.6 to 2.8 ± 1.9 in the NR group. However, at the last follow‐up, the VAS score of chest pain in the OR group was statistically lower than that in the NR group (1.4 ± 1.0 *vs* 2.1 ± 1.6, *P* < 0.05).

### *Grades of Surgical Outcomes for Cervical Angina*

The majority of the patients’ cervical angina symptoms were relieved postoperatively (Table 4). In the OR group, the results of cervical spine surgery for cervical angina were as follows: excellent grade in 18 patients (85.7%) and fair grade in 3 patients (14.3%). In the NR group, there were 10 patients (50.0%) of excellent grade, 9 patients (45.0%) of fair grade, and 1 patient (5.0%) of in poor grade. Notably, there were statistically significant differences between the two groups (χ^2^ = 6.265, *P* = 0.044), which indicated that patients with resection of Luschka's joint osteophyte obtained better clinical improvement of chest pain. No major complications such as graft migration collapse, displacement, or neurological impairment occurred during the follow‐up time. The average follow‐up was 32 months (26–52 months) in the OR group and 29 months (24–49 months) in the NR group, respectively.

**TABLE 4:** Results of cervical spine surgery for cervical angina in two groups (cases, %)

Groups	Excellent	Fair	Poor
OR group	18 (85.7)	3 (14.3)	0 (0)
NR group	10 (50)	9 (45)	1 (5)
Pearson χ^2^‐test	χ^2^ = 6.265, *P* = 0.044*		

* Means statistically significant difference.

NR, the nonresection; OR, osteophyte resection.

**Fig. 3:**
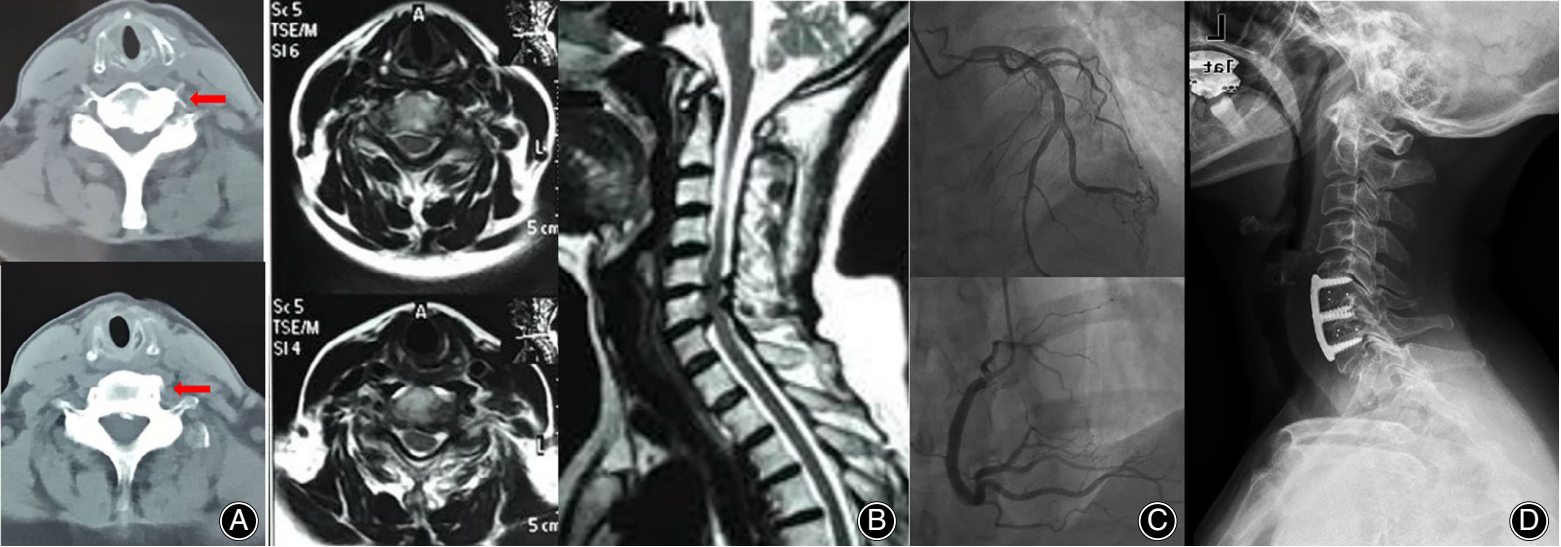
A case of a 47‐year‐old woman. MRI images show two segments (C_5–6_ and C_6–7_) of cervical disc hernia and neuroforaminal stenosis; the Luschka's joint osteophyte located at C_6–7_ level (A, B). Coronary artery angiography showed no artery stenosis (C). Anterior cervical discectomy and fusion (C_5–7_) surgery with resection of Luschka's joint osteophyte (D).

An example case is shown in Fig. 3. This is a 47‐year‐old woman who presented with paroxysmal anterior chest wall pain for 1 year, associated with neck pain radiating down her left arm. Over the preceding 6 months, the symptom were exacerbated and could not be relieved with nitroglycerin or bed rest. She had no past medical history of coronary heart disease. During the current hospitalization, cardiac workups were all negative results. Coronary artery angiography showed no left or right coronary artery stenosis. Neurological examination revealed positive Spurling maneuver. MRI images showed two segments (C_5–6_ and C_6–7_) of cervical disc hernia and neuroforaminal stenosis; the Luschka's joint osteophyte was located at C_6–7_ level. She underwent anterior cervical discectomy and fusion (C_5–7_) surgery with resection of Luschka's joint osteophyte. After the operation, her angina symptoms were soon relieved and this benefit was maintained during 28 months of follow‐up.

## Discussion

In 1927, Phillips^21^ first described the symptomatic patients with anginoid pain due to cervical nerve root compression. Nachlas^3^ in 1934 reported three cases of precordial pain arising from cervical spine conditions, which responded to traction and physical therapy. Other early classical studies^22^, ^23^, ^24^ also reported cases of precordial pain attributed to cervical arthritis or herniated cervical discs. In 1950, Davis^25^ first proposed “cervical angina” as a clinical syndrome. In recent publications^1^, ^2^, ^4^, ^6^, ^8^, investigators followed the diagnosis name of “cervical angina.”

### *Diagnostics* *of Cervical* *Angina*

Although cervical angina presents in varied ways, there are some certain features in the patient's history and physical examination that should increase suspicion for cervicogenic chest pain. The pain was often induced by cervical range of motion or movement of the upper extremity, usually persisting for >30 min or less than 5 s^26^. Up to 50% to 60% of patients experience autonomic symptoms such as dizziness, nausea, diaphoresis, and sympathetic nervous signs^2^, ^8^. Once coronary artery disease has been adequately excluded, cervical imaging can be helpful in defining anatomic abnormalities^27^. Plain radiographs or MRI may demonstrate degenerative cervical changes, including disc space narrowing, osteophyte formation, or neuroforaminal encroachment. In our series, all patients underwent consultation with cardiologist and extensive cardiac evaluations (electrocardiogram, cardiac enzymes test, cardiac stress testing, and coronary angiograms if necessary) to adequately exclude coronary artery disease. Their radiographic evidence of degenerative changes in the cervical spine support the diagnosis of cervical angina.

### *Mechanism of Chest Pain*

The physiological etiology of cervical angina remains unclear. Early studies believed ventral motor root compression as responsible for the “protopathic” pain, referring to the same area as the distribution of the affected nerve^3^. In a retrospective study of 438 cases, Brodsky *et al*.^2^ indicated that 70% of cases with cervical angina have been attributed to cervical nerve root compression. Radicular pain is likely mediated by compression of the C_4_ to C_8_ nerve roots, which supply the sensory and motor innervation to the anterior chest wall through the medial and lateral pectoral nerves. LaBan *et al*.^28^ supported this theory and presumed that the spinal cord ventral roots produced protopathic pain around the chest. Cervical myelopathy could also be the origin of precordial pain, independent of root compression. According to Nakajima's observation^8^, left lower chest pain tended to appear as a radicular sign, while retrosternal, epigastric pain, and autonomic symptoms tended to be accompanied with myelopathy. In the myelopathy cases, autonomic symptoms were more evident than upper arm symptoms. Lesions of the dorsal horn or disruption of the ascending cardiac spinothalamic tracts may create the sensation of anginoid pain^11^, ^29^. More importantly, based on these theories, previous studies have proved that symptoms of cervical angina can be improved following cervical surgeries with decompression of the spinal canal and the neural foramina^2^, ^4^, ^8^, ^9^.

In our series, we found an interesting phenomenon that patients with cervical angina demonstrated the hyperplasia of Luschka's joint osteophyte in their degenerative cervical vertebrae. These osteophytes were located at lower cervical regions: C_6_–C_7_ (19 cases, 46.3%), C_5_–C_6_ (17 cases, 41.5%), and C_4_–C_5_ (4 cases, 12.2%). In our opinion, there are two reason for this phenomenon. First, as a natural feature of spinal degeneration, the Luschka's joint osteophytes usually occur in the lower cervical region. Second, from the anatomic view, osteophytes on the upper cervical region may be too high up for sympathetic contributions to chest pain. The average area of Luschka's joint osteophyte was 34.85 mm^2^ and the average length were 5.09 mm. On MRI images, due to the compression of the Luschka's joint osteophyte, the area of the homolateral longus colli was smaller compared to the contralateral longus colli. This is an underestimated radiological characteristic that has never been fully recognized in cervical angina. We hypothesize that the Luschka's joint osteophyte and its compression on homolateral longus colli may play a role in cervical angina. To evaluate the potential role the Luschka's joint osteophyte, the patients were randomly divided into two groups. After anterior cervical decompressive surgery, the majority of the patients’ cervical angina symptoms were relieved postoperatively (with a total of 28 cases of excellent grade and 12 cases of fair grade). However, by comparison, patients who underwent the resection of Luschka's joint osteophytes achieved better results than those without resection of Luschka's joint osteophytes. In addition, in the OR group, postoperative VAS scores for the chest were relatively lower and were maintained at the last follow up.

**Fig. 4:**
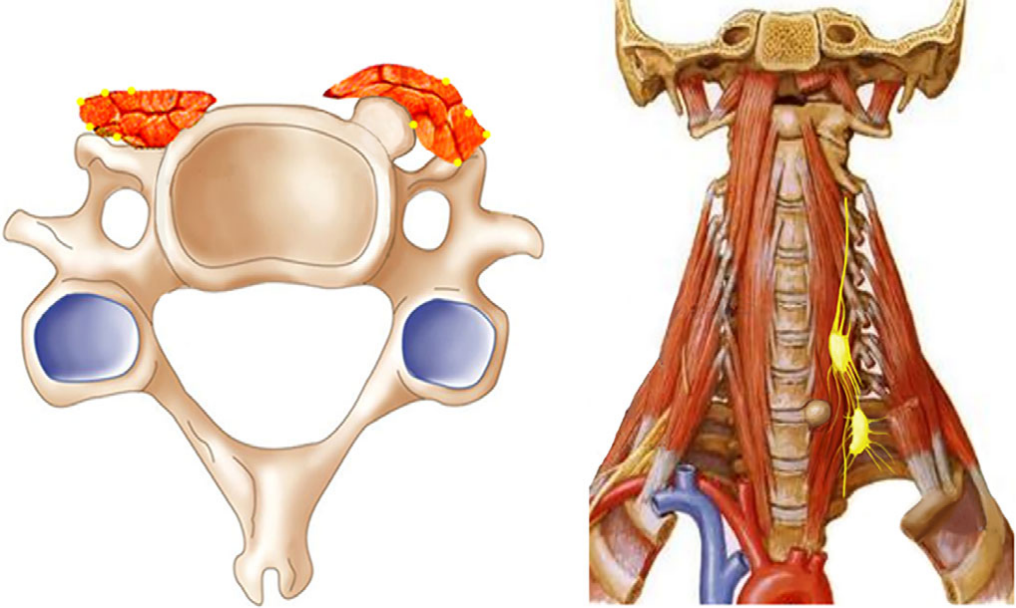
The cervical sympathetic chain is located laterally to the longus colli. Luschka's joint osteophyte compressed the homolateral longus colli in a bowstring shape, which might stimulate the adjacent sympathetic chain, resulting in angina symptoms.

These findings indicated that anterior cervical decompressive surgery with resection of Luschka's joint osteophyte could effectively reduce angina symptoms and improve the quality of life. Besides nerve root compression, Luschka's joint osteophyte may be another pathogenic factor in cervical angina. Sympathetic afferent fibers lie on the anterolateral side of the cervical vertebrae. The Luschka's joint osteophyte is in close proximity to the sympathetic afferent fibers to the heart and coronary arteries. The protrusion of Luschka's joint osteophyte jacks up the homolateral longus colli, like a bowstring effect, which might compress the adjacent sympathetic nerve (Fig. 4). It is reasonable to speculate that perturbation of the sympathetic nervous system may result in noncardiac chest pain. Moreover, the anatomy of the cervicodorsal spine and the adjacent nerve structure reveals the following pertinent facts: the medial anterior thoracic nerves originating in the eighth cervical and first thoracic spinal segments and the lateral anterior thoracic nerve originating in the sixth and seventh cervical segments innervate the pectoralis major and pectoralis minor muscles. The thoracalis longus and thoracodorsalis of the subscapularis nerve begin in the roots of the fifth, sixth, seventh, and eighth cervical segments and supply the teres major and teres minor, the subcapsularis and the deeper portions of the latissimus muscle. The Luschka's joint osteophyte and its bowstring effect may irritate the aforementioned nerve passage, which is a possible anatomic integration of cervical angina symptoms.

### *Limitations*

Some limitations of this study should be addressed. First, the cervical angina sample size was small due to its nature of low incidence. The strict inclusion criteria also limited the sample to those patients with both clinical and radiographic findings of cervical angina. Second, to focus on the potential role of Luschka's joint osteophyte, we did not include cervical angina patients without Luschka's joint osteophyte in this study. Finally, this study is based mostly on the details of clinical observation in the medical records. Further pathological study should be explored in Luschka's joint osteophyte in patients with cervical angina.

### *Conclusion*

In conclusion, this is the first prospective study focusing on this unusual presentation of cervical pathology; namely, cervical angina. Based on the results of the current study, anterior cervical decompression surgery with resection of Luschka's joint osteophyte can effectively reduce angina symptoms and improve patients’ quality of life. In addition to nerve root compression, Luschka's joint osteophyte may be another pathogenic factor in cervical angina.
